# Developing local crisis leadership – A research and training agenda

**DOI:** 10.3389/fpsyg.2023.1041387

**Published:** 2023-02-02

**Authors:** Jarle Eid, Anita Lill Hansen, Natalia Andreassen, Roar Espevik, Guttorm Brattebø, Bjørn Helge Johnsen

**Affiliations:** ^1^Center for Crisis Psychology, University of Bergen, Bergen, Norway; ^2^Department of Psychosocial Science, University of Bergen, Bergen, Norway; ^3^Centre for Research and Education in Forensic Psychiatry, Haukeland University Hospital, Bergen, Norway; ^4^Business School, Nord University Business School, Bodø, Norway; ^5^Department of Leadership and Command & Control, Swedish Defence University, Stockholm, Sweden; ^6^Department of Anaesthesia and Intensive Care, Haukeland University Hospital, Bergen, Norway; ^7^Department of Clinical Medicine, University of Bergen, Bergen, Norway

**Keywords:** crisis leadership, complex problem solving, simulation, team training, technology, performance, resilience, emergency

## Abstract

The crisis triggered by Covid-19 has exposed the interdependencies of modern society and sparked interest in local response to protracted and complex crisis situations. There has been a growing awareness and interest in the key roles of political and professional stakeholders, their emotional regulation and how they influence team performance and outcomes in dealing with uncertainty and complex crisis situations. While cognitive and behavioral aspects of crisis leadership are well researched, less is understood about how one can mitigate negative emotions, instill trust, or restore public faith and support of security forces and emergency response teams during crises. In addressing this gap, we propose a simplified conceptual roadmap for research and training of local crisis leadership. In this, we emphasize *complex problem solving*, *team interaction, team context and technology* and *team training design*. These four factors represent significant barriers if neglected. On the other side, they may be considerable force multipliers when better understood and managed. We suggest how seven research and training questions could be linked to the four conceptual factors and guide an evidence-based approach to develop local crisis leadership.

## 1. Introduction

Crises can generally be understood as rare events that are unexpected, highly salient, and potentially disruptive for individuals, organizations, and societies (Bundy et al., 2017). Still, crisis events can also become crucial turning points for positive change and provide new learning opportunities when they are well managed (James et al., 2011). Success or failure in crisis situations rests on the training and preparedness of frontline personnel and local crisis response units. The protracted response to the Covid-19 pandemic emphasized the significance of political leaders as part of the crisis response in local communities. Local political or senior administrative leaders can serve an important role, by instilling trust and assure the public about societal preparedness and safety. The pandemic showed that political leadership matters: from international cooperation, or the lack thereof, to the influence on individual behavior, the nature of politics shapes responses and outcomes (O’Flynn, 2021). Even if local resources are limited and need to be complemented by regional or national resources, the local communities often have to shoulder the main efforts in the initial crisis response. Still, there is limited research on training and development of local crisis response capacities, procedures, and interoperability. Thus, the overall aim of the present study is to contribute to close this gap by suggesting an evidence-based framework for research and training of crisis leadership and resilience management in local municipalities.

The uncertainties, disruptive, and cascading effects of Covid-19 prompted a need to shift from risk management to resilience management (Pescaroli and Needham-Bennett, 2021). One of the core issues of resilience management is the local capacity to deliver safety critical services to the public, despite uncertainty and disruption. In general, resilience management will refer to the political and operational capability to respond, adapt, recover, and learn from, disruptive and threatening events. Not surprisingly, the pandemic has spurred a renewed interest in the emotional aspects of crisis leadership (Wittmer and Hopkins, 2021). The emotional regulation of the leader – follower exchange works both ways and influence trust, resilience, and team performance in complex crisis situations (Gooty et al., 2010). Leaders’ capability to respond and adapt to a high-risk situation, including their self-awareness and emotional awareness may influence task coordination, communication, and performance at the team level (Dasborough and Scandura, 2021). In a review of crisis leadership studies Bavik et al. (2021) concludes that while studies of cognitive and behavioral aspects of crisis leadership is common, less is understood about how leaders can mitigate negative emotions and restore the positive emotions of stakeholders and constituencies during crises.

Covid-19 reinforced the crucial role of local crisis leadership by highlighting the interdependence and need to coordinate organizational assets to ensure an effective response (Cheng, 1983) from municipality leaders. The pandemic highlighted the need for close collaboration between public health officials and political leaders to work in concert over time to address the immediate, long-term, and recurring consequences from the pandemic. In many communities’ business executives were forced to consider layoffs (Oen et al., 2022). In the public sector school closures and distance learning raised new challenges for school principals (Lien et al., 2022). Subsequently, there is a growing awareness about the need to consider policy implications of protracted crisis interventions, educate, train, and prepare local municipality leaders for their roles in a crisis response. In addressing this gap, we propose a multidisciplinary framework and offer eight research questions to enhance our understanding of how individual, interpersonal, and contextual factors contributes to local crisis leadership. We believe training of local crisis response units needs to be informed by an interdisciplinary approach based on operational psychology (Johnsen et al., 2010), organizational dynamics (Weick and Quinn, 1999; Salas et al., 2005), and political science (Christensen et al., 2019) addressing both the human, operational, structural and policy aspects of crisis leadership. In addition, the Covid-19 pandemic has highlighted the special challenges of protracted situations where contextual factors influence crisis leadership, trust, and resilience over time (Pescaroli et al., 2021).

To this end, we allude to four core assumptions. First, in line with Dörner and Funke (2017), we believe there is a need to build better and more systematic knowledge about emotional aspects of complex problem solving since decision making represents a core aspect of crisis leadership and team processes. Crisis leadership in protracted and cascading events taxes heavily on limited cognitive and emotional resources. Madera and Smith (2009) examined how the expression of anger and sadness influences the evaluation of leaders in crisis situations. Their study revealed that a leader expressing sadness was evaluated more favorably than a leader expressing anger. Managing emotions is therefore a vital element of crisis leadership and complex problem solving skills before, during, and after emergency situations (Jordan and Troth, 2004).

Second, we follow up Flin et al. (2017) emphasis on the need to address the interpersonal dynamics in operational assessment, training, and preparedness. It is hardly surprising that crisis leadership in emergency services is highly dependent on teamwork (Salas et al., 2007a). Teams are identifiable social work units consisting of two or more people with several unique characteristics including: (1) dynamic social interaction with meaningful interdependencies; (2) shared and valued goals; (3) a discrete lifespan; (4) distributed expertise; (5) clearly assigned roles and responsibilities (Salas et al., 2007b). Thus, local crisis leadership units need to consider how to develop and regulate both positive emotions (i.e., trust, collective efficacy, confidence) and negative emotions (i.e., frustration, anger, fear) that are necessary for creating resilient high-performance teams (Salas et al., 2007a). Even if cognitive, emotional, and interpersonal skills are seen as crucial, these “non-technical skills” have often been ignored or taken for granted, since leaders in crisis response units are mostly recruited based on their professional seniority and technical skills.

In local crisis situations decision making and coordination of activity will flow through the normal chain of command using emergency dispatch centers or directly through an operational staff (Dörner and Funke, 2017). The latter depends on the complexity, sustainability, and consequences of the situation where it is anticipated that the resources of the dispatch centers will be exceeded. However, both coordinating entities rely heavily on a seamless coordination of individual capabilities, team resources, and available structural assets to mobilize a sustained crisis response. Still, coordination through “the chain of command” and by operational staff, presents different levels of complexity. A better understanding of how the command-and-control processes operate in local crisis leadership could therefore be important to improve performance and design more effective unit-level training programs (Zemba et al., 2019).

Third, we emphasize the need to consider how contextual factors such as cascading or protracted events and technological failure may influence crisis leadership, risk, and disaster preparedness (Pescaroli et al., 2021). Effective crisis leadership depends on close inter-agency coordination and collaboration at the local, regional, and national level to maintain a resilient response. A particular situation will define the needs of a response operation, the roles and procedures that can be applied and the conditions for teamwork, constituting team context. To fully understand and develop effective crisis leadership in the municipalities, we believe there is a need to also understand the nature of external contingencies. Contextual factors, such as organizational environment, technological equipment and use of coordination and control tools, and available information on the situation, will constitute challenges for the response, but also represent opportunities to develop relevant training and skill assessment for local crisis response units.

Fourth, state-of-the-art simulator environments have been adopted to improve training and allow scenario based, true to life training in crisis leadership (Roberts et al., 2021). Simulations may offer an excellent opportunity to train non-technical skills in a controlled, safe, and realistic environment (Saus et al., 2010). Different types of simulation exercises may be a useful tool to train stakeholders who deal with crises and develop their skills on making decisions under stress, dealing with own emotions, situation, and their team. A resilient response rests on the capacity of front-line operators and stakeholders to uphold their mission, despite setbacks and critical stressors (i.e., technological breakdown of strategic infrastructure). The total spectrum of human factors, from individual complex problem solving *via* dynamics in team and team-training and the availability of normative structural guidance, is crucial to crisis leadership. However, focus of training is often on testing and following procedural guidelines. More information is still needed on how emotional factors and team dynamics could enhance training and development of local crisis response units (Bavik et al., 2021).

**Figure 1:**
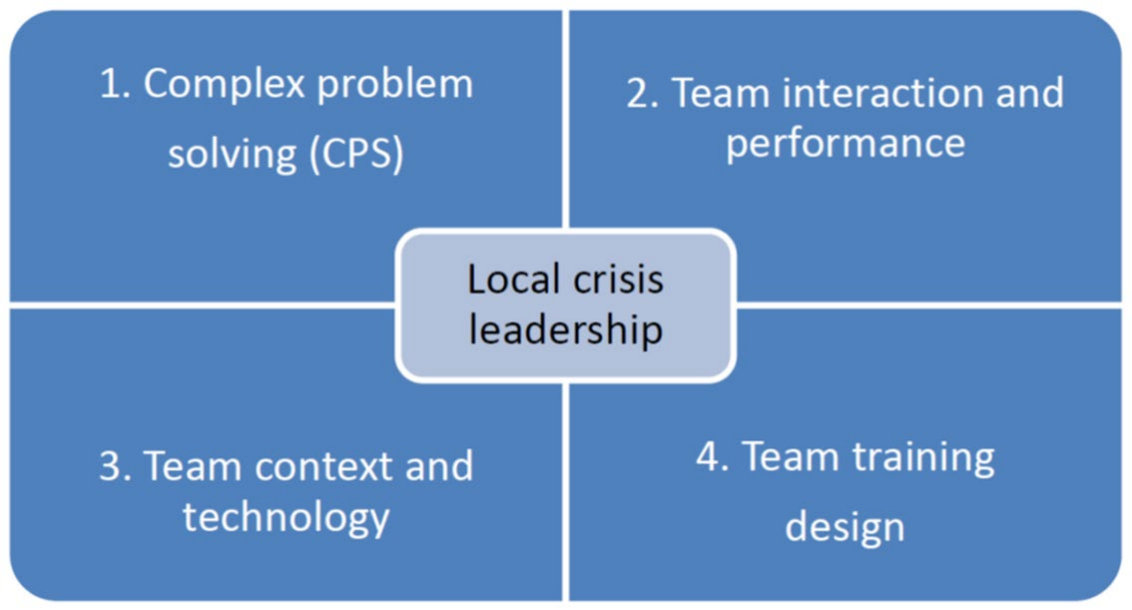
Core aspects of local crisis leadership.

As depicted in Figure 1, we propose a generic and multidisciplinary approach to understand local crisis leadership as a function of psychological, interpersonal, and contextual factors. These factors may represent significant barriers or considerable force multipliers, depending on how they are understood and managed. While each factor is evidence based and can inspire future research, we believe this generic and relatively simple model will be easy to understand and apply to team training design and development of local crisis leadership capacities across different crisis response units. In the following, the model will be outlined in more detail.

## 2. Complex problem solving

Complex problem solving is an integrated aspect of crisis leadership. Complex problem solving can be regarded as a collection of self-regulated psychological processes and activities necessary in dynamic environments to achieve diffuse or ill-defined goals that cannot be reached by routine actions (Dörner and Funke, 2017). For example, the problem solving processes of a local crisis response leader influenced by cognitive (e.g., judgment, decision-making, planning abilities), emotional (e.g., regulation of emotions elicited by a situation), and motivational aspects (e.g., material, or ecological rationality) (Sayegh et al., 2004). These cognitive and emotional phenomena are of particular importance in high stakes situations where risks are interconnected, interacting, or cascading (Pescaroli and Alexander, 2018). A study by Zemba et al. (2019) suggested that research on complex problem solving must focus more on the original complexities of real-life problems to improve our understanding of how humans deal with pressing problems. Although machine learning is developing rapidly, human involvement in decision processes will still play a significant role in the foreseeable future (Roberts et al., 2021). Since humans cognitively appraise and react emotionally to environmental cues in situations (Gross, 2002), it is therefore of particular importance to train and develop local crisis leadership skills.

In general, acute stress and crisis usually elicit negative emotional states such as fear and worry. Understandably, a lack of systematic training and preparedness of political or administrative leaders, poses a risk of introducing an extra element of uncertainty to complex problem solving in local crisis leadership. Based on the classical understanding of decision-making and rationality, emotions are seen as irrational and negatively related to higher order cognitions such as judgement and decision-making (Kahneman, 2011). However, the relationship between emotions and rationality is complex, since emotions also may have a positive impact on decisions (Pham, 2007). For example, Hansen et al. (2009) reported that fear induction augmented the cognitive performance in military personnel. An important issue in crisis leadership is therefore how local crisis response leaders appraise their emotional reactions to stressful and rare events. In short, how they regulate and make use of their affective responses in safety critical situations (Gyurak et al., 2012). In local crisis response negative emotions such as frustration, anger, or fear may have a significant disruptive effect on the quality of crisis leadership (Jordan and Lindebaum, 2015). From this we propose the following research and training objective:

1: How will negative emotion induction influence the outcome of complex problem solving in local community responses to crisis situations?

Although there is consensus that affective states will influence complex problem solving, research indicates differential outcomes depending on the emotion regulation strategies used (Ford and Mauss, 2015). Research has shown that there are cultural differences in emotion regulation strategies for instance, individuals from Asian backgrounds are more likely to use suppression compared to individuals with a European background (Ford and Mauss, 2015). Recent research has therefore started to investigate the effects emotions and emotion regulation may have on different practical domains in life (e.g., Ford and Gross, 2018; Karnaze and Levine, 2018). Still, research investigating crisis leadership’s choice of emotional regulation strategies is scarce, and even more so, how local political leaders attend to the emotional aspects of protracted crisis situations. Great variation exists in selection, education, and training of crisis response personnel, from no selection and very little training of political/strategic decision makers to extremely strenuous procedures for selection and training of specialized crisis response units. This in turn may contribute to cultural differences between local crisis response units and crisis managers. A pressing issue is therefore if potential variations in emotional regulation strategies may have adaptive consequences in strategic, tactical, and operational approaches to an unfolding crisis? From this, we believe it will be of importance to investigate the effects of negative emotion induction and to identify the choice of emotion regulation strategies across different crisis response entities.

Moreover, to prepare local decision-makers for future crises, evidence-based training and development need to be designed and implemented to improve crisis leadership (Lacerenza et al., 2018). Complex problem solving usually involve knowledge-rich requirements and collaboration among subject matter experts with different expertise and backgrounds, including novices. Thus, more knowledge about complex problem solving on the individual level will have important implications for team interaction and performance, team context and technology, as well as team training design. To this end we propose the following research and training objective:

2: How can emotion regulation strategies be useful in complex problem-solving situations that taxes different levels of cognition?

## 3. Team interaction and performance

A local crisis response unit can be a group of two or more individuals who have specific roles and interact adaptively, interdependently, and dynamically toward a common and valued goal (Roberts et al., 2021). Thus, an understanding of how situation awareness in individuals, teams and systems are impacted and evolve are important in developing local crisis response capacities, teamwork, and training. For an overview of research on situational awareness (see Salmon et al., 2008; Stanton et al., 2017). Teamwork is essential for effective crisis response in emergency services and law enforcement. Teamwork can be defined as the knowledge, skills, and attitudes critical for team members to interdependently interact with one another effectively in such a way that leads to positive team-based outcomes (Salas et al., 2009). Thus, the local crisis response is dependent on skilled individuals who can perform effectively as a collective. In many instances operational situations will require new, creative, or highly skilled coordination of actions in time critical situations (Lacerenza et al., 2018). Studies of Naval operations have shown that just one new team member may have significant disruptive effects on team interaction and effectiveness (Espevik et al., 2011). To maintain a resilient response over time, local crisis response units need to train, coordinate, and develop their taskwork and teamwork resources from various professionals.

The Shared Mental Model (SMM) approach (Salas et al., 2005) emphasizes the importance of shared knowledge structures within a team to facilitate coordination and performance. The model consists of three coordinating mechanisms (trust, shared mental model, and closed loop communication) to ensure even distribution of information within the team. These coordinating mechanisms further guide the five team-processes of leadership, monitoring, support, adaptation, and team orientation (Salas et al., 2005). Thus, the performance of a local crisis response unit will depend on the flow of information from the coordinating mechanisms and the outcomes of the team processes. A core aspect of SMM is the ability to predict other team members actions. There is a link between the SMM and complex problem solving in that the mental model includes a shared history of affective experiences from significant gains and losses in previous team interaction. This joint history of previous actions contributes to the SMM and facilitates the prediction of other team-members’ affect and future behavior (Thornton and Tamir, 2017). A better understanding of team dynamics involved in local crises response will then serve to develop future crisis leadership capacities and more effective team training. Thus, our third research and training objective is as follows:

3: How can coordinating mechanisms and team processes enhance situational awareness and quality of local decision making in an interdisciplinary crisis response staff?

Frontline policing is an example of first echelon crisis leadership where the incident commander relies heavily on communication with the police dispatch center, which acts as an information hub and coordinating entity, cooperating closely with the emergency medicine and critical care, fire and rescue operational centers. The goal is to save lives and preserve health in time critical situations. In most cases the incident commander will coordinate through the line of command, but if the crisis is perceived as too massive or requires a protracted response, an operational staff is established (i.e., coordination through staff). Both coordination through the line and staff involves team-behavior, since a team is defined as two or more subjects coordinating their activities toward a common goal (Parelta et al., 2018). One promising strategy to increase team performance in operational situations has been to develop greater NTS awareness in policing (Schaveling et al., 2017). In operational situations there is therefore a need to identify, explain, and mitigate dysfunctional and functional NTS both in the form of normal coordination “through the line” and in the more complex coordination through a crisis response staff when managing protracted societal emergencies (Rico et al., 2008).

Shared mental models is in general considered to facilitate team coordination and support to accommodate team performance over time and in response to changing demands in the environment (Mathieu et al., 2000). Still, anecdotal evidence and preliminary studies (Terpstra, 2018) of crisis leadership have indicated a lack of situational awareness, flexibility and procumbent quality of decisions when coordinating through staff compared to coordinating through the line. Since the organizational set-up is similar for police, health, and the fire & rescue services a generic model could be developed with relevance for all emergency services. However, the coordination between professional crisis responders and the local political or administrative leaders presents additional challenges. Thus, an in-depth understanding of the cognitive, emotional, and behavioral workings of front-line response teams would be crucial to command and coordinate local resources. From this, we suggest the following research and training objective:

4: How will mechanisms and processes of coordination influence local crisis response when coordinating is facilitated by staff versus by line?

While studies on teamwork traditionally have focused on face-to-face contact, recent studies have also examined virtual teams (i.e., geographically dispersed operators). A challenge for operational virtual teams is that the teamwork processes become technology dependent. Although less studied in virtual teams, a promising theoretical approach to develop teamwork processes is the “shared mental models” (SMM) approach to sensemaking in teams (Rafaeli et al., 2009). Overlapping mental models within a virtual team enables individual team members to anticipate or predict the activities and needs of other team members (Cannon-Bowers et al., 1993). Research from our group suggests that SMM contributes to enhance performance in police units (Espevik et al., 2021). However, a common factor in these studies was the significance of face-to-face contact within teams. In virtual teams, sub-optimal team performance is often caused by process loss due to a failure to synchronize the mental models of individual members (Rico et al., 2008).

In a review Peavey and Cai (2020) concluded that decentralized intensive care units were less likely to call for support due to a lack of visibility and proximity between caregivers. A recent study investigated medical dispatch teams (operators and ambulances) using the theoretical framework of SMM (Johnsen et al., 2022). Although an acceptable fit was found for the theoretical model, the different elements varied in their predictive power. This indicates a need to provide a better and more comprehensive understanding of distributed team processes. Floren et al. (2018) argue that the SMM construct itself lacks clear operational definition and conclude in their review that surprisingly few studies describe educational interventions aimed at SMM development or attempt to measure the construct. To close these gaps, we propose the need for empirical studies that could describe and explain coordinating mechanisms and team processes in front-line operators and how team processes in distributed teams will contribute to improve situational awareness and decision making in a local crisis response staff. Typically, a local operational staff will include seasoned subject matter experts (personnel, intelligence, operations, logistics, planning etc.). Notably, these local organizational units are rarely mustered and/or trained. Rotation of personnel, infrequent training, and limited use of operational staff in operational situations represents a significant barrier to establishing shared cognitive structures and sufficient training to facilitate effective team performance. To our knowledge there is limited knowledge about the use and effectiveness of such shared knowledge structures in a local operational staff compared to teams comprised of dispatch operators and patrols. Still, the use of virtual teams has increased in many domains during Covid-19 and could present a preview of future ways to organize and train operational staff functions. The increased potential for information exchange through technological aids could facilitate, impair, or have a neutral effect on effectiveness (Johnsen et al., 2019). Most studies on the development of SMM and its relation to team effectiveness are anchored in an understanding that the relation is motivated by increased interaction between team members, increased communication, and training. In general, a reasonable assumption would be that more use of information communication technology could augment the effect of human interaction processes (i.e., teamwork) on team effectiveness. In this domain few if any studies have investigated the effect of both coordinating mechanism and team-processes on crisis management staff in virtual teams. To close this gap, there is a need to examine differences in coordinating mechanisms, team processes and effectiveness between virtual and face-to-face staff work based on the SMM framework. Thus, our fifth research and training objective is as follows:

5: Can the SMM framework explain possible differences in coordinating mechanisms, team processes and effectiveness between virtual and face-to-face coordination of local crisis response?

## 4. Team context and technology

Crisis leadership and team performance are influenced by contextual factors (Andreassen et al., 2020). Following from Oc (2018), context can be seen at two different levels: 1) the omnibus context and 2) the discrete context. In a crisis such as the COVID-19, the omnibus context involves a broad consideration of contextual or environmental influences such as health care resources, economy, political stability, and socioeconomic factors. In contrast, the discrete context relates to specific situational variables that influence behavior directly or moderate relationships between variables in a specific context. Thus, according to Oc (2018) crisis leadership and team processes will be influenced by context in a nested manner, with the discrete context subsumed within the omnibus context.

To maintain a resilient local response to crisis situations, public and private resources need to coordinate procedures and maintain backup plans and redundancies. While organizational routines can both be seen as a source of inertia and inflexibility, they can also be an important source of adaptability and change in crisis situations (Feldman and Pentland, 2003) by guiding behavioral responses. Still, vulnerabilities in many sectors are indirectly associated with dependencies on the specific routines and technologies in use. An example of this is how Norwegian school principals encouraged and succeeded in a rapid transformation to remote learning and digital education during COVID-19 (Lien et al., 2022). A better understanding of how high reliability in infrastructure and organizational routines could influence the behavior and practices of crisis managers during technological breakdown and cascading risks will provide a significant advantage for crisis leadership (Pescaroli and Alexander, 2018).

Compounded, interconnected, interacting, and cascading risks could have a profound effect on critical societal functioning (Pescaroli and Alexander, 2018). Further research is needed to provide a better understanding of how contextual factors will influence crisis managers and how the Sendai Framework for Disaster Risk Reduction could inform disaster risk reduction (Aitsi-Selmi et al., 2015). The operational capacity of local crisis responses in remote geographical areas is increasingly dependent on the reliability of technologies such as internet-based services or satellite systems (Andreassen et al., 2020). In crisis situations, technological breakdown or disturbances can prompt a need to shift to other means of interaction, incl. Low-tech intensive coordination forms. A better understanding of technological limitations, failures and consequences will present important information on factors that could affect the information sharing and coordination capacity of crisis leadership in remote areas such as the High North (Andreassen et al., 2020). Local knowledge and trust in the crisis response units will be important to mobilize local resources and initiative. The prospect of technological failure could present an important element in training and development of local crisis response units to enhance mental readiness, training fidelity, and interoperability of leaders and local resources (Hays and Singer, 1989). To this end we propose the following research and training objective:

6: How will technological failures affect inter agency coordination and preparedness to cope with local crisis situations?

## 5. Team training design

A cornerstone in crisis response involves flexible response to threats, which encompasses a capacity of shifting plans. The flexibility is influenced by individual risk perceptions, skills, competencies, and the resolve of local crisis managers. Selection, training, and organizational culture could influence both risk perception and willingness to take risk. For instance, Johnsen et al. (2016) concluded that a brief eight-hour training session of frontline police officers increased their awareness of risks associated with critical decisions and intentions to act if a critical situation should occur. An interesting finding was that the training effects were more pronounced for unexperienced compared to seasoned police officers. Future research should therefore contribute to identify and explain perceived gaps in preparedness and risk assessment when local crisis leadership is exposed to technological failures.

Following from the above, a next step is to investigate mitigating factors derived from real world crisis situations. Since training studies, including simulator training, are characterized by a high degree of control, there is a need for studies of real-life operations to examine crisis leadership in action in true to life situations with high degree of uncertainty, flexibility, and affect. To gain a better understanding of the contextual aspects of crisis leadership, a potential roadmap to enhance crisis leadership could be to explore different forms of training designs to mitigate the impacts of technological failures including relevance of training, subjective learning effects, change in awareness and potential for behavior change.

Team development interventions is commonly used to describe activities aimed at improving team effectiveness, processes, or skills (Lacerenza et al., 2018). Crisis leadership in remote and complex situations such as in the Arctic, requires local knowledge and skills to successfully operate in an environment with scarcity of resources and infrastructure, multiple independent actors, and limited situation awareness (SA). The coordination processes are commonly planned and formalized as standard operating procedures, mechanisms, or task assignments in position descriptions. The importance of coordination in fast-response organizations has been acknowledged because of the involvement of interdisciplinary teams of specialists and distributed operations (Faraj and Xiao, 2006). Still, lack of resources, technological failures, or various local contextual factors, may challenge standard operating procedures developed for normal situations.

In complex environments the application of formalized plans presents coordination challenges. Crisis situations may call for formal coordination based on analytical decision-making processes but may also invoke improvised coordination mechanisms to maintain flexibility due to task complexity or irrational components in decision making, such as negative emotions. Training is a primary tool for enhancing the competence of local responders to coordinate their actions when faced with uncertainty or emotionally charged decisions. Evidence based team training interventions adopt a structured (i.e., step by step) approach to developing the relevant knowledge, skills, and attitudes that underlie effective teamwork (Lacerenza et al., 2018). To enhance local team leadership and collective performance, one can use structured simulation and training exercises to investigate how team behavior and leadership adapts to a dynamic situation in training or in real-life situations (Lacerenza et al., 2017).

Simulation-based training is widely considered the most effective way of delivering team training (Weaver et al., 2010). Simulation-based training is recognized to improve knowledge, technical skills, and behavioral learning (Cook et al., 2011). Not surprisingly, simulation-based activities have the strongest evidence of effectiveness (Buljac-Samardzic et al., 2020). Technologies in a digital exercise could be set up in a way so that even geographically separated team members may collaborate in a learning session. Remote, but coordinated digital training exercises provide an easy and cost-effective way to assess, train, and develop local crisis response units. The participants’ experiences contribute to the task practice environment that may affect the enhanced team coordination in real practice. Advanced simulation technology provides multiple opportunities for developing team coordination skills and competences under realistic circumstances. Participants may receive feedback on their performance, increase their awareness of team dynamics and individual stress reactions (Meynard and Gilson, 2014). For instance, information communication technology (ICT) can be used to connect stationary and virtual teams to demonstrate how task and team based sheared mental models will be positively associated with outcomes in virtual teams (Meynard and Gilson, 2014). Another desirable outcome is that a simulated and recorded exercise will allow identification and evaluation of role acceptance, role flexibility, and decision-making (Wilson et al., 2009). However, one of the main challenges in simulation training is to design specific situations relevant to actual practice and to capture the complexity of real-world incidents in the training experiences (Flin, 1996). An aspect of real-life crisis situations will also be to deal with contingencies, side effects, and the consequences of unforeseen emergencies (Alharthi et al., 2018).

Thus, it is important to plan pedagogies and study whether the technology employed has provided richer interactions between learners, their concepts, and practice (Laurillard, 2008). A promising pedagogical approach to team simulation scenarios would be to utilize an event-based measurement approach that is anchored to a relevant local crisis. Through the systematic introduction of events into training exercises, event-based measurements provide opportunities to observe and learn from specific team and leadership behaviors that occur in response to the event (Fowlkes et al., 1998), for instance to foster diagnostic reasoning, patient management, and practice for surgery in training of physicians.

Teamwork in local crisis management entities could be the passkey to assess and establish a collective understanding in support of a seamless coordination of performance solutions to complex scenarios. However, following the notion that human interaction is central, it also represents a vulnerability factor in crisis management. Therefore, empirical, and systematic evaluation of team training to improve local team behavior is essential to develop successful crisis leadership and a resilient community response. Following a deeper understanding of cognitive and emotional aspects of decision making and team processes, it will be possible to empirically investigate training effects of known individual and team processes affecting crisis response outcomes (Saus et al., 2012).

Flexibility is an example of a core aspect of both individual and team level crisis response. Flexibility in crisis response demands the coordinators to fulfill a wide range of roles related to information sharing, decision-making, and front-end personal command. Role flexibility and role transitions create challenges to crisis responses units. In complex crisis situations team members self-regulatory processes are important (DeShon and Rench, 2009) and role expectations in the form of role acceptance and improvisation will be a key factor in crisis leadership, team coordination and SMM.

During the response process, incident commanders will coordinate and control the situation through specified routines according to their roles, standard operating procedures or tasks lists within the established incident command systems (Bigley and Roberts, 2001). On-scene coordination of a complex emergency response operation will rely on coordinators that fulfil a range of roles related to information sharing, decision-making, and front-end resource command. Understanding of their own and other roles is essential for crisis leadership, team coordination and SMM.

Roles are defined as a set of expectations in connection to a position or fulfilment of an assignment, for instance in fulfillment of responsibilities in security operations (Bartone, 2010). If role expectations are contrary to one’s personal or to fellow team members, this may cause intrapersonal or interpersonal conflicts. In unforeseen situations, a role shift by a team member may not be explicitly shared by the team, hampering teamwork processes, or resulting in role flexibility and improvisation (Michaelsen and Sweet, 2008). There is a need to explore how a role improvisation can be defined in terms of role expectations and acceptance and how to handle differences in role acceptance and improvised role shifts.

In acute situations, when operational staff are established, effective crisis leadership may implicate the need to shift roles to facilitate desired outcomes. Role switching is one of the coordination mechanisms for high reliability organizing that involves the reassignment of personnel to different positions within the organization in complex and dynamic environments (Summers et al., 2012). To evaluate and enhance the awareness of team dynamics, effective crisis leadership will rest on collaborative team simulation and training through table-top and simulator exercises. The information, communication technology used to support team interaction and collaboration in emergency scenarios has the inherent capacity to enhance crisis leadership by training of team effectiveness, role acceptance and flexibility for both virtual and face to face situations. Advanced simulation technology provides opportunities for developing NTS under realistic circumstances. Technologies for digital tabletop exercises may also involve virtual teams. From this we propose a need to investigate the challenges facing crisis leadership in achieving shared understanding of team roles and designing team training to support front-end operators working in distributed teams.

Since the availability of on-site simulator training is often very limited, and user costs are high, individual computer-based training may serve as a useful supplement to full scale team training (Saus et al., 2010). In any case, effective feedback and assessment are necessary to enhance training outcomes (Marcano et al., 2019). Simulation and training that focus on coordination of information and expert knowledge among members of a team is an essential part of exercising crisis leadership in complex emergencies. There is a need to explore the most effective strategies that responders with various background can employ in training to enhance the flexibility of a command system. From this we propose the final research and training objective:

7: How can emergency management exercises be designed to enhance role understanding and coordination flexibility in response to local crisis situations?

## 6. Summary

The current paper presents a simplified conceptual roadmap and suggests research questions that could inform, develop, and improve local crisis leadership. Following up from Bavik et al. (2021) we have indicated knowledge gaps in our understanding of how human factor variables and “soft skills” facilitate individual, interpersonal, and inter-agency performance in complex, acute, and protracted crisis situations. Extending previous research on non-technical skills and operational psychology, we allude to a multidisciplinary approach informed by theoretical perspectives from organizational, behavioral, and political science, that can be informed by a mixed methods approach including experimental and qualitative approaches applied to simulation, training, and real-life situations. From a generic model of crisis leadership, we propose seven research questions that can inform local training and development programs. Hence, we propose to examine the full spectrum of crisis response, from individual complex problem solving to frontline team dynamics and assess how local crisis leadership and teamwork respond, to technological breakdowns and contextual risk factors (Roberts et al., 2021).

We strongly believe that a multi-disciplinary approach will contribute significantly to generate new theoretical and applied knowledge. From a theoretical perspective there is a need to gain more knowledge about how local professional and political leaders are influenced by their emotions during crisis situations, and how these emotion regulation strategies influence complex decision making. At the interpersonal level, new theoretical perspectives on team dynamics, role acceptance and role improvisation need to be compared to existing theory of shared mental models in true to life simulation and locally relevant training scenarios. A part of this is to develop evidence-based team training procedures that will serve an applied purpose in assessing and developing frontline operators in public and private sectors. At the policy level there is a need to provide evidence-based guidelines to support political decision-makers in dealing with diffuse and uncertain scenarios, producing new guidelines for stress testing organizations and improving resilience to known and unknown threats. The research themes summarized in Table 1, suggest relevant research questions that can inform the training and development of local end-users. An improved understanding of individual and team-based problem-solving capacity in emotionally charged situations will feed back to improve education, training, and performance in frontline operators.

**Table 1:** Topics and research questions to extend the empirical basis for assessing and developing local crisis leadership.

Local crisis leadership	Research questions
Complex problem solving	How will negative emotion induction influence the outcome of complex problem solving in local community response to crisis situations? How can emotion regulation strategies be useful in complex problem-solving situations that taxes different levels of cognition?
Team interaction and performance	How can coordinating mechanisms and team processes enhance situational awareness and quality of local decision making in an interdisciplinary crisis response staff? How will mechanisms and processes of coordination influence local crisis response when coordinating is facilitated by staff versus line? Can the SMM framework explain possible differences in coordinating mechanisms, team processes and effectiveness between virtual and face-to-face coordination of local crisis response?
Team context and technology	How will technological failures affect inter agency coordination and preparedness to cope with local crisis situations?
Team training design	How can emergency management exercises be designed to enhance role understanding and coordination flexibility in response to local crisis situations?

## 7. Outcomes and consequences

We expect that competent community-based crisis leadership will have a positive impact on the general public’s perception of professionalism and competence, thus instilling trust in the emergency services. A better understanding of how local crisis response units attends to complex problem solving and emotional regulation in operational settings, may also provide better advice and recommendations to the general population in safety critical situations (Dasborough and Scandura, 2021). Likewise, by acquiring more empirical information about team interaction and performance this will present an opportunity to communicate best practice advice about barriers and facilitating factors involved in functional team behavior (Roberts et al., 2021). From enhanced team training we expect the explicit focus on simulation and training to be of high relevance for local public and private sector organizations that increasingly rely on distributed work teams, simulations, and online solutions to train their employees or solve their core mission assignments. Thus, this simplified conceptual framework will also have an impact on societal preparedness by providing an empirical basis for selection, education, and training of crisis management teams. Finally, a possible radical outcome of a stronger emphasis on crisis leadership could be a change in the relation between operational dispatch centers and operational front-end operators. This could support a shift from risk management to a stronger emphasis on resilience management (Pescaroli and Needham-Bennett, 2021). A more explicit focus on local crisis leadership could involve more specific performance expectancies, changes in role expectancies as well as increased role acceptance and a renewed scrutiny of training practices and the dynamics of effective crisis leadership.

## Author contributions

JE: writing-reviewing and editing, conceptualization, and supervision. AH: writing-original draft preparation, and conceptualization. NA, GB, and RE: writing-original draft preparation. BJ: writing-reviewing and editing, conceptualization. All authors contributed to the article and approved the submitted version.

## Conflict of interest

The authors declare that the research was conducted in the absence of any commercial or financial relationships that could be construed as a potential conflict of interest.

## Publisher’s note

All claims expressed in this article are solely those of the authors and do not necessarily represent those of their affiliated organizations, or those of the publisher, the editors and the reviewers. Any product that may be evaluated in this article, or claim that may be made by its manufacturer, is not guaranteed or endorsed by the publisher.
